# Implication of Retrobulbar and internal carotid artery blood-flow-volume alterations for the pathogenesis of non-arteritic anterior ischemic optic neuropathy

**DOI:** 10.1186/s12886-021-02075-2

**Published:** 2021-08-25

**Authors:** Zhiyong Fu, Hongyang Li, Yanling Wang

**Affiliations:** Department of ophthalmology, Beijing Friendship Hospital, Capital Medical University, Beijing, 100050 China

**Keywords:** Blood flow volume, Optical coherence tomography angiography, Pathogenesis, Non-arteritic anterior ischemic optic neuropathy

## Abstract

**Background:**

To analyze blood flow volume alteration that involved both retrobulbar artery and internal carotid artery (ICA) in patients with non-arteritic anterior ischemic optic neuropathy (NAION) and to assess their relevance for the pathogenesis of NAION.

**Methods:**

Forty two patients with NAION (unilateral affected) and 42 age-matched controls participated in this study. By head-and-neck computed tomographic angiography (CTA), the diameter of ICA and ophthalmic artery (OA) were measured. By colour Doppler imaging (CDI), the mean blood flow velocity (Vm) and the blood flow volume of ICA and OA were measured or calculated. By optical coherence tomography angiography (OCTA), peripapillary and optic disc vessel density were measured. Data obtained from the affected side of the patients were compared to those of the contralateral healthy side and the control.

**Results:**

Compared with the controls and the contralateral healthy side of the patients with NAION, the diameter of ICA, the blood flow volume of ICA and OA, the peripapillary and optic disc vessel density in the affected side decreased significantly (*p* < 0.05). However, there was no statistical difference in the diameter of OA (*p* = 0.179, 0.054 respectively), the Vm of OA (*p* = 0.052, 0.083 respectively), or the Vm of ICA (*p* = 0.364, 0.938 respectively) between groups. Peripapillary and optic disc vessel density were significantly positive correlated with the blood flow volume in ipsilateral ICA and OA in patients with NAION (all *p* < 0.01).

**Conclusions:**

The reduction of blood flow volume was more prominent in OA and ICA than decrease of Vm, peripapillary and optic disc vessel density were significantly positive correlated with the blood flow volume of ipsilateral ICA and OA in patients with NAION.

**Supplementary Information:**

The online version contains supplementary material available at 10.1186/s12886-021-02075-2.

## Background

Non-arteritic anterior ischemic optic neuropathy (NAION) is among the leading causes of optic nerve impairment in the middle-aged and elderly [1, 2]. Numerous clinical and experimental studies have been conducted in patients with NAION, which suggested that there was early affection of retrobulbar and carotid artery haemodynamics alterations in the course of the disease [3–6]. Previous study demonstrated that NAION patients showed markedly different retrobulbar haemodynamics with reduced circulation velocity in ophthalmic artery (OA) measured by colour Doppler imaging (CDI) [4]. Furthermore, some researchers suggested that NAION might occur simultaneously with stenosis and reduced blood flow of internal carotid artery (ICA) [7, 8]. Due to these reasons, we believed that it was important to assess OA and ICA blood flow while studying the pathogenesis of NAION. Because the siphon region of ICA (ICAS) is often implicated in ocular ischemic diseases and the changes of blood flow here might affect the lower reaches’ hemodynamics directly [9], ICAS was designated as the anatomical site for the study of ICA when it was involved.

With the emergence of optical coherence tomography angiography (OCTA), the study of microvasculature of the posterior pole has made great improvement, and this technique sets up a bridge for understanding the relationship between distal macrovascular blood flow volume variations and the developing of NAION. In the present study, we hypothesized that the changes in peripapillary and optic disc vessel density correlate with the blood flow volume alterations in OA and ICA, and aiming to illustrate the importance of evaluating retrobulbar artery and ICA blood flow volume in patients with NAION.

## Methods

A total of 42 patients with unilateral NAION were included in this study, comprising 27 male and 15 female, aged 47—77 years. The mean interval between onset of NAION and measurement was 5.2 ± 2.7 days. All patients met the diagnostic criteria of NAION, including sudden painless loss of visual acuity; presence of optic disc edema shortly after the disease onset; optic disc-related visual field defects; and an absence of other ocular diseases that might affect the optic nerve [10, 11]. All patients were clinically diagnosed with NAION after consultating from neurology, infectious diseases, cardiology and rheumatology, in order to exclude any systemic or neurologic diseases that might affect the optic nerve. Patients were excluded if they had serious media opacities or had undergone previous intraocular surgery. The control group consisted of 42 age- matched subjects with no history of chronic ocular disease or of ocular surgery, comprising 25 male and 17 female, aged 49—74 years. Participants were excluded if they had a history of chronic corticosteroids, aspirin, or related drugs use.

This study was approved by the Ethics Committee of Beijing Friendship Hospital affiliated to Capital Medical University and was performed in accordance with the Declaration of Helsinki, and informed consent was obtained from all participants.

**Fig. 1:**
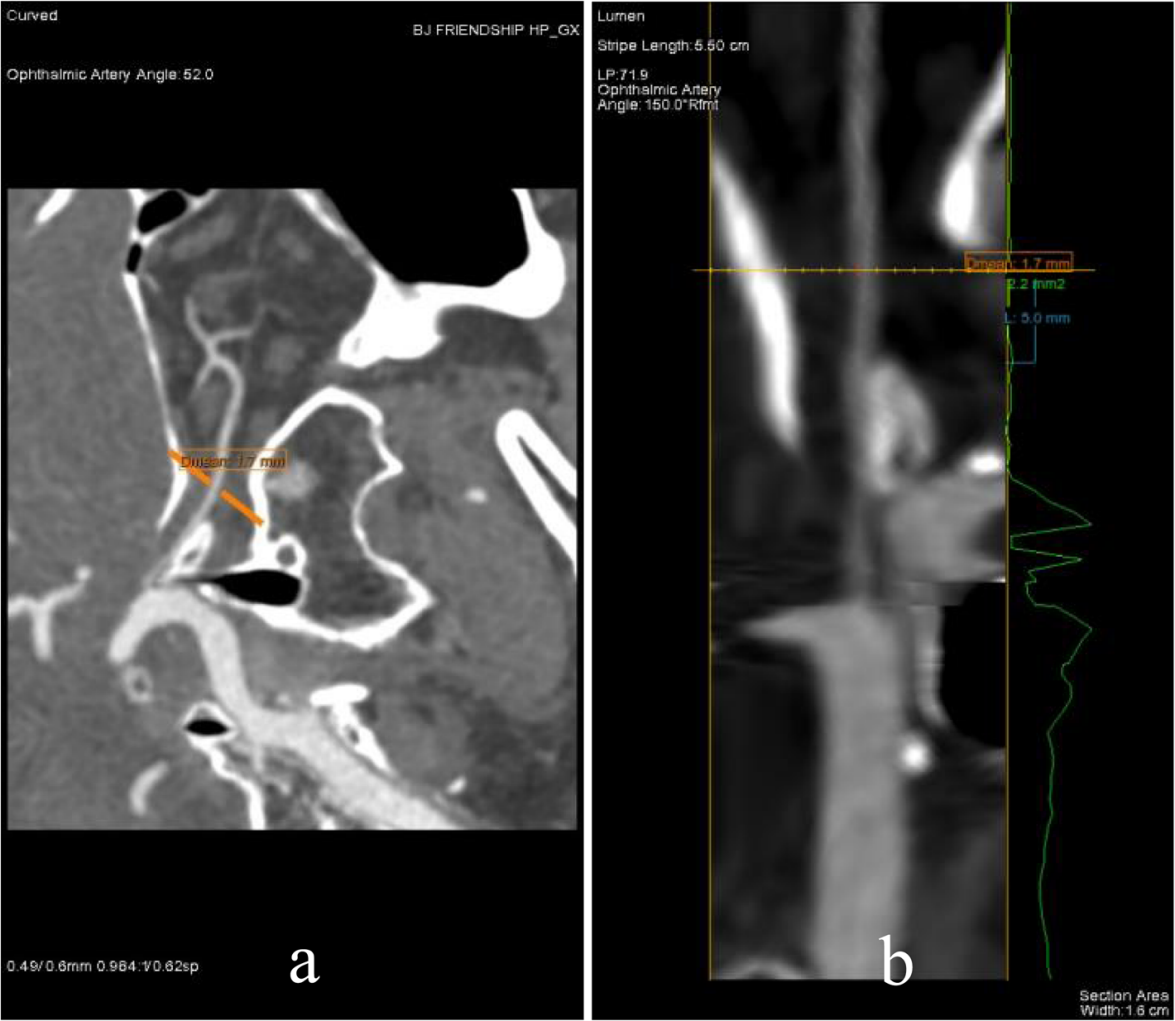
Head-and-neck CTA inspection. Showed the diameter of OA (**a**, **b**)

Ocular data were collected from all participants, including slit-lamp Ophthalmoscopy, fundus examination, non-contact intraocular pressure (IOP) measurement, fundus photography and spectral-domain optical coherence tomography scans (SD-OCT, Heidelberg, Germany). The Vm of OA and ICA were measured by CDI, and the diameter of OA and ICA were measured by CTA as previously described [12, 13]. The diameter of OA was measured by selecting the blood vessel 5 mm above the level of optic canal outlet and measuring its diameter of (Fig. 1).

**Fig. 2:**
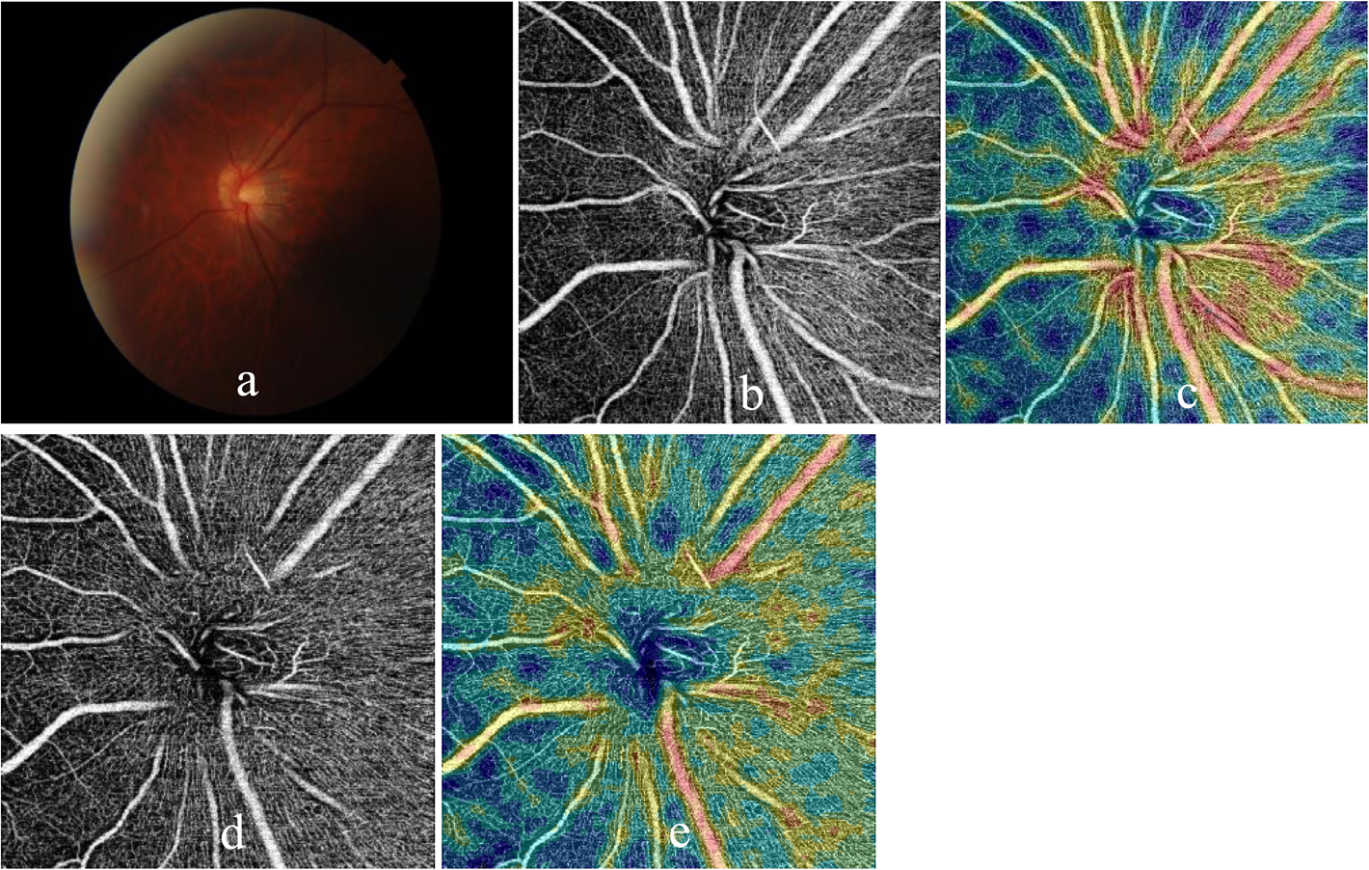
OCTA examinations of peripapillary and optic disc vessel density of a control subject (left eye). Color fundus photograph (**a**),vessel density in the peripapillary retina on nerve head mode (**b**, **c**) and optic disc on RPC mode (**d**, **e**) were included (**b**, **d**: images in gray scale, **c**, **e**: color-coded images)

**Fig. 3:**
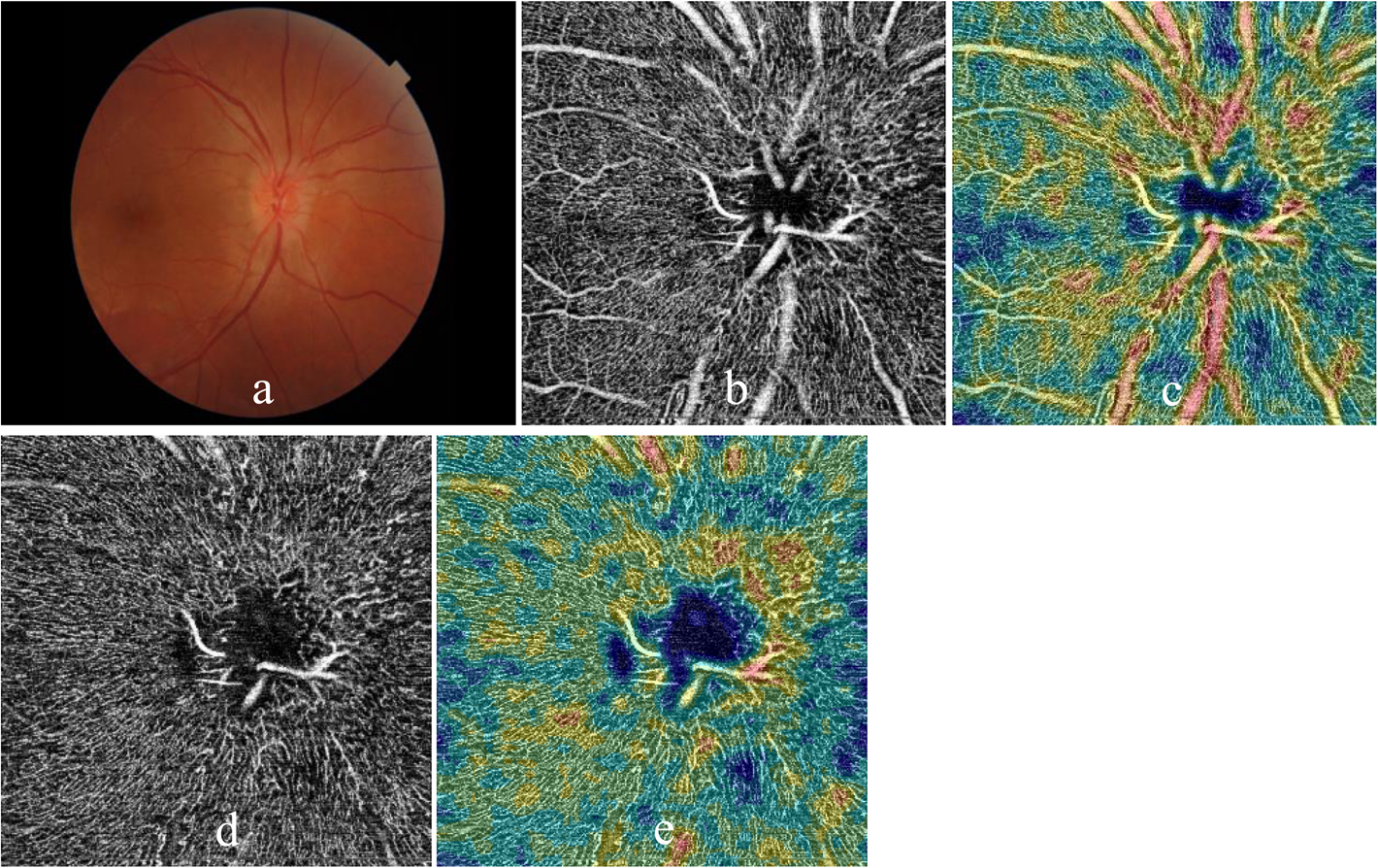
OCTA examinations of peripapillary and optic disc vessel density of a NAION patient (affected left eye). Color fundus photograph (**a**),vessel density in the peripapillary retina on nerve head mode (**b**, **c**) and optic disc on RPC mode (**d**, **e**) were included (**b**, **d**: images in gray scale, **c**, **e**: color-coded images)

Thirty of 42 patients and 30 of controls also underwent OCTA (OCT-A, Optovue, USA) examination after CDI examinations. The OCTA images were acquired using the Optovue Avanti, and two imaging windows (4.5 × 4.5 mm and 6 × 6 mm) were obtained. OCTA examination was employed to evaluate peripapillary and optic disc vessel density. Among the four references the system provides, the nerve head mode and RPC mode were used in the present study. The nerve head mode included signals from the inner limiting membrane (ILM) to 150 mm below the membrane, and the RPC mode included signals from ILM to nerve fiber layer (NFL) (Figs. 2, 3).

The blood flow volume (A) of ICA and OA was calculated using the following formula:

$$ \mathrm{A}=\uppi {\mathrm{r}}^2\times \mathrm{Vm}\left(\mathrm{r}=1/2\kern0.5em \mathrm{diameter}\right) $$

Blood pressure (BP) was determined by sphygmomanometry in a sitting position after a rest of 10 min.

All the measurements were performed by experienced operators unaware of the subject’s condition. The left side measurements were defined as the results of each control.

Statistical analyses was conducted via the SPSS statistical package (SPSS, V. 21.0, Chicago, IL, USA). The Kolmogorov–Smirnov test was used to determine the distribution pattern of all obtained data. Continuous variables were compared between groups using two- sample t-test and categorical variables were analyzed by χ2 test. Pearson correlation coefficient analysis were used to evaluate the associations between peripapillary and optic disc vessel density with the blood flow volume of ICA and OA. The values were presented as means±standard deviation (SD) and were considered statistically significant at *P* < 0.05.

## Results

Concerning the demographic and clinical data of the NAION patients and the controls, the age, gender composition, IOP, systolic BP (SBP), diastolic BP (DBP) and the proportion of systemic diseases showed no statistically significant difference between two groups(*p* > 0.05) (Table 1).

**Table 1:** Demographic and clinical data of the patients with NAION and controls

Groups	NAION	Controls	*P*
Age (years)	61 ± 8	60 ± 7	0.544*
Gender, no. of male (%)	27 (64.3%)	25 (59.5%)	0.503**
IOP (mm Hg)	16 ± 3	15 ± 3	0.264*
SBP (mm Hg)	138 ± 23	131 ± 18	0.134*
DBP (mm Hg)	77 ± 11	73 ± 9	0.077*
Hypertension,n (%)	13 (31.0%)	10 (23.8%)	0.268**
Diabetes mellitus,n (%)	11 (26.2%)	10 (23.8%)	0.744**
Hyperlipidemia,n (%)	20 (47.6%)	15 (35.7%)	0.086**
Ischaemic heart diseas,n (%)	5 (11.9%)	3 (7.1%)	0.228**

Independent-samples t-test *, χ2 test **, Paired- samples t-test ***

*NAION* non-arteritic anterior ischemic optic neuropathy, *IOP* intraocular pressure, *SBP* systolic blood pressure, *DBP* diastolic blood pressure

Compared between the affected side and the contralateral healthy side in the patients with NAION, the diameter of ICA, the blood flow volume of ICA, the blood flow volume of OA, peripapillary and optic disc vessel density all significantly decreased (*p* < 0.05). There was no statistical difference in the diameter of OA (*p* = 0.054), the Vm of OA (*p* = 0.083), or the Vm of ICA (*p* = 0.938) (Table 2).

**Table 2:** Comparison of diameter, Vm, blood flow volume of OA/ICA and vessel density between the affected and the contralateral healthy side in the patients with NAION

Groups	NAION eye	Normal eye	*P*
OA diameter (mm)	1.39 ± 0.12	1.41 ± 0.14	0.054***
OA Vm (cm/s)	11.56 ± 2.77	12.17 ± 2.75	0.083***
OA blood flow volume (mm^3^/s)	176.96 ± 56.67	193.00 ± 61.04	0.015***
ICA diameter (mm)	2.90 ± 0.72	3.18 ± 0.50	0.001***
ICA Vm (cm/s)	46.04 ± 5.83	45.97 ± 5.52	0.938***
ICA blood flow volume (mm^3^/s)	3097.75 ± 1131.55	3691.07 ± 1076.24	< 0.001***
Peripapillary vessel density (% area)	48.90 ± 6.02	56.33 ± 2.58	0.001***
Optic disc vessel density (% area)	45.77 ± 4.91	54.75 ± 2.39	0.001***

Independent-samples t-test *, χ2 test **, Paired- samples t-test ***

*NAION* non-arteritic anterior ischemic optic neuropathy, *OA* ophthalmic artery, *Vm* mean blood flow velocity, *ICA* internal carotid artery

Compared between the affected side of NAION and the controls, the diameter of ICA, the blood flow volume of ICA, the blood flow volume of OA, peripapillary and optic disc vessel density all significantly decreased (*p* < 0.05). There was no statistical difference in the diameter of OA (*p* = 0.179), the Vm of OA (*p* = 0.052), or the Vm of ICA (*p* = 0.364) (Table 3).

**Table 3:** Comparison of diameter, Vm, blood flow volume of OA/ICA and vessel density between the affected side of the patients with NAION and the controls

Groups	NAION eye	Controls	*P*
OA diameter (mm)	1.39 ± 0.12	1.43 ± 0.15	0.179*
OA Vm (cm/s)	11.56 ± 2.77	12.52 ± 1.45	0.052*
OA blood flow volume (mm^3^/s)	176.96 ± 56.67	200.79 ± 41.65	0.031*
ICA diameter (mm)	2.90 ± 0.72	3.36 ± 0.51	0.001*
ICA Vm (cm/s)	46.04 ± 5.83	47.03 ± 3.92	0.364*
ICA blood flow volume (mm^3^/s)	3097.75 ± 1131.55	4196.79 ± 1095.75	< 0.001*
Peripapillary vessel density (% area)	48.90 ± 6.02	57.08 ± 3.02	0.001*
Optic disc vessel density (% area)	45.77 ± 4.91	55.51 ± 2.72	0.001*

Independent-samples t-test *, χ2 test **, Paired- samples t-test ***

*NAION* non-arteritic anterior ischemic optic neuropathy, *OA* ophthalmic artery, *Vm* mean blood flow velocity, *ICA* internal carotid artery

Concerning the correlation between the blood flow volume and vessel density in the affected side of patients with NAION, we found both peripapillary vessel density and optic disc vessel density were significantly positive correlated with the blood flow volume in ipsilateral ICA and OA (all *p* < 0.01) (Table 4).

**Table 4:** Correlation analyses between vessel density and the blood flow volume (affected side of the patients with NAION)

Parameters	Peripapillary vessel density		Optic disc vessel density	
	*r*	*P*	*r*	*P*
OA blood flow volume	0.516	0.004	0.631	< 0.001
ICA blood flow volume	0.527	0.003	0.578	0.001

*OA* ophthalmic artery, *ICA* internal carotid artery

## Discussion

NAION is believed to be the consequence of acute ischaemia of the optic nerve head [14], but the exact pathogenesis of NAION remains a matter of debate. The occurrence of NAION is the result of the interaction of many factors, and as we have known, the presence of ischemic ocular tissue reduces demand for blood flow is one of the explanations for the onset of NAION. On the other hand, through the exploration of the ICAS region configuration of NAION patients, with the help of different examination instruments, we realized that the blood flow changes caused by narrowing of this region, with resultant ischemia to the optic nerve head, is more likely to contributes to the pathophysiology of NAION. As mentioned earlier, previous studies suggested that the alterations in OA and ICA blood flow were often involved in ocular ischemic diseases [4, 7, 8]. Therefore, the present study attempted to establish a link between blood flow volume alterations in retrobulbar artery and ICA and the pathogenesis of NAION.

With the development of medical imaging technology and examination methods, more and more research tools are available to us. CDI is an ultrasonic imaging modality that provides a display of the real-time direction and velocity of the blood flow imposed over a conventional gray-scale B-mode ultrasound image [15]. Previous studies found that patients with NAION showed reduced circulation velocity in the retrobulbar vessels and decreased velocities of blood cells in the capillaries of the optic nerve head by means of CDI or laser Doppler velocimetry [4, 5, 16, 17], but the resolution of CDI is insufficient to provide reliable volumetric flow measurements. Fortunately, it’s possible to detect the diameter of blood vessels using CTA precisely. Nowadays, CTA has been recommended as a safe and non-invasive tool for the assessment of OA, carotid, coronary, and renal artery stenosis due to its ability to achieve clear image reconstruction [18–20]. With the data obtained from CDI and CTA, blood flow volume of ICA and OA can be calculated indirectly according to Vm and blood vessel diameter. In this study, the OA diameter 5 mm before the outlet of optic canal was selected for measurement based on the following two considerations: 1. Special anatomical characteristics of OA; 2. Clear anatomical marks on CTA. As the first major branch of the internal carotid artery, the OA passes through the optic canal in the dural sheath, accompanied by the optic nerve. Generally, the OA emits the first branch at 10–15 mm retrobulbar. Therefore, there is no branch of OA 5 mm before the outlet of optic canal, and the diameter measured here can represent the average diameter of OA in the orbit before branch. In addition, the two sphenoid winglets have obvious humps on CT images, which can be used as clear anatomical markers to accurately locate the measurement points.

In our study, we found the diameter of the ipsilateral ICA distinctly narrowed in the affected side of patients suffering from NAION, compared with the contralateral healthy side or with the controls. Accordingly, the blood flow volume of ICA in the patients with NAION significantly decreased, although there was no significant difference in Vm of ICA between groups. As a rule, the flow velocity increases as a result of constriction or narrowing of a vessel lumen [21]. However, Considering that blood is a non-Newtonian fluid, the viscosity of plasma and the elasticity of the vessel wall both have an effect on the flow velocity [12]. According to the law of conservation of mass, when blood flows through a certain section of blood vessel, the blood flow volume should be a fixed value, so we can obtain the instantaneous blood flow volume by measuring the diameter of the vessel and the blood flow velocity. We speculated that when NAION occurred, decreased intravascular blood flow was caused by narrowing ICA diameter, which ultimately resulted in significantly reduced blood perfusion to the microvessels in the downstream region. However, previous studies proved that the degree of ICA stenosis had no significant differences between the NAION group and the controls [22–24]. This discrepancy might be explained by the different measurement locations selected. In previous studies, the entire ICA vessel was taken as the research object, while in this study, only the siphon segment of ICA was selected as the measurement object. We designed it this way because ICAS are considered to be a very specific part of the ICA vascular pathway, and the tortuous configuration of ICAS is crucial to diseases related to turbulent circulation [25], especially to ocular ischemic diseases.

In addition, we noted that the diameter of OA and the Vm of OA were not significantly different in NAION patients compared with the contralateral healthy side and the controls, but the blood flow volume of OA in NAION patients was significantly reduced compared with the other two groups. Combined with the measurement results of ICAS, we believed that the blood flow velocity alone is not a reliable parameter in the study of NAION, while the blood flow volume seems to be more reliable, which can accurately reflect the status of blood perfusion. Based on these findings, it was reasonable to assume that evaluating blood flow is more important in ischemic diseases than focusing solely on blood flow velocity.

To further investigate the relationship between blood flow volume alterations of OA and ICA and the developing of NAION, we measured peripapillary and optic disc vessel density. Moreover, the correlation between vascular density and blood flow volume were analyzed. Currently, laser speckle flowgraphy (LSFG) and OCTA have both been developed as non-invasive methods for evaluating ONH blood flow, and they are both reliable and repeatable techniques [26–29]. LSFG is a promising tool for the evaluation of microvascular lesions in the retina and ONH, and further clinical validation is required [25, 26]. In this study, OCTA was used to quantitatively evaluate the microcirculation changes of ONH in patients with NAION. The reason for this choice was that, on the one hand, previous studies have described potential efficacy for OCTA in the evaluation of NAION through quantified detecting reduced ONH perfusion [30–32], and on the other hand, it was reported that vessel densities at the peripapillary retinal and optic disc were closely related to retinal nerve fiber damage and visual field loss [33, 34], and OCTA is able to display both structural and blood flow information to help quantify the ONH perfusion and retinal vasculature [35, 36]. In our study, the result showed that peripapillary and optic disc vessel density all significantly decreased in the affected side of NAION patients compared with the contralateral healthy side and the control group, which is consistent with previous studies [30, 31]. Furthermore, we found both peripapillary vessel density and optic disc vessel density were significantly positive correlated with blood flow volume of ICA and OA in the affected side of patients with NAION. Peripapillary and optic disc vessel density can be used as a reliable and stable indicator for the changes in peripheral vascular status caused by OA and ICA blood flow volume alterations. However, back to the pathogenesis of NAION, we must be sober in making conclusions, because correlation does not imply causation. ICA siphon region narrowing, with resultant ischemia to the optic nerve head, could contribute to the pathophysiology of NAION, but it could be a result of the NAION rather than a cause. Considering other explorations for the pathogenesis of NAION, what we have done is to incorporate the blood vessels outside the retrobulbar vessels and the latter into the field of study, and the focus of research is the alternation of blood volume instead of blood velocity. Hopefully, the efforts we have made will provide some potential possibilities for the study of NAION, and the panorama will become more complete and clear with detection techniques improving and explorations deepening someday.

There are several limitations in our study. Firstly, the selection bias of participants might potentially affect the results. To minimize this bias, we designed age- and sex-matched controls, and attempted to make sure that the distribution of vascular disease characteristics was matched between patients and controls. However, Since systemic diseases are more common in the study group (although the differences of prevalence of systemic diseases between patients and controls were not statistically significant), it was still difficult to select a control group on a per- patient basis during the recruitment process. Larger scale studies with better case-control matching would be required. Secondly, the presence of an optic disc edema can induce artifacts as measured by OCTA, and it’s challenging to visualize the peripapillary network clearly. But we believed that the error range was acceptable because we had eliminated all the unsatisfactory images in the present study, and we attempted to verify our conclusion using multimodal vascular imaging techniques in our future study. Another limitation of this study is that the blood flow volume obtained here was only an approximation, which was calculated based on a formula rather than directly detected by an inspection equipment. Further prospective blood flow volume studies with more accurate calculations or detection methods are necessary to confirm the present conclusions in study of NAION patients.

## Conclusions

In summary, the reduction of blood flow volume was more prominent in OA and ICA than decrease of Vm, peripapillary and optic disc vessel density were significantly positive correlated with the blood flow volume of ipsilateral ICA and OA in patients with NAION.

## Supplementary Information

**Additional file 1.**

## Data Availability

All data generated or analysed during this study are included in this published article [and its supplementary information files].

## Abbreviations

ICA: Internal carotid artery
NAION: Non-arteritic anterior ischemic optic neuropathy
CTA: Head-and-neck computed tomographic angiography
OA: Ophthalmic artery
CDI: Colour Doppler imaging
Vm: Mean blood flow velocity
OCTA: Optical coherence tomography angiography
ICAS: Siphon region of ICA
IOP: Intraocular pressure
SD-OCT: Spectral-domain optical coherence tomography scans
ILM: Inner limiting membrane
NFL: Nerve fiber layer
BP: Blood pressure
SBP: Systolic blood pressure
DBP: Diastolic blood pressure
LSFG: Laser speckle flowgraphy
