# Supplementary material for: Implication of Retrobulbar and internal carotid artery blood-flow-volume alterations for the pathogenesis of non-arteritic anterior ischemic optic neuropathy
Source: BMC Ophthalmol. 2021 Aug 25;21:309. doi: 10.1186/s12886-021-02075-2 (PMC8390251; doi:10.1186/s12886-021-02075-2)
Supplement: Supplementary file 1 — Additional file 1. [file 12886_2021_2075_MOESM1_ESM.docx]

**supplementary data part 1**


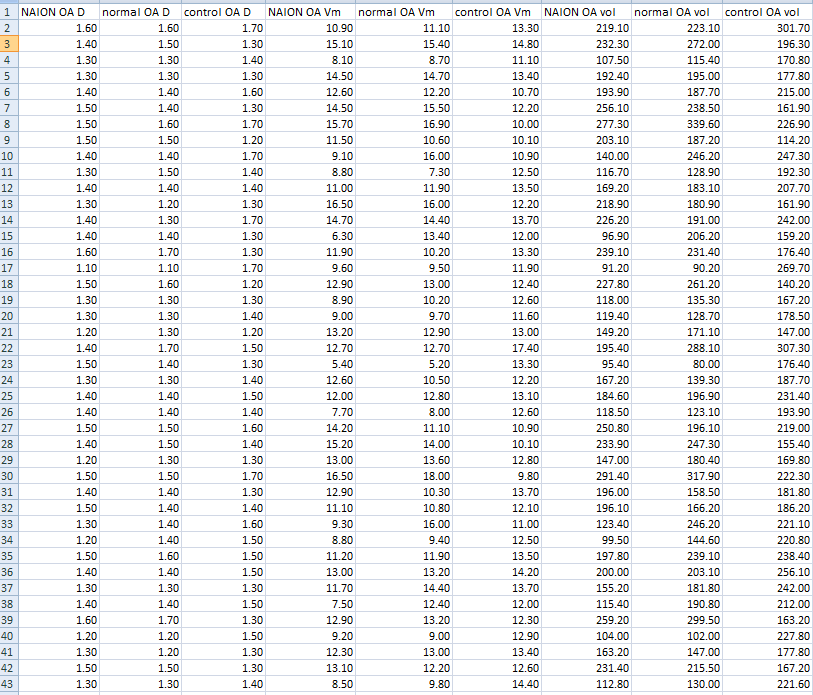


*NAION OA D* the diameter of ophthalmic artery (NAION eye), *normal OA D* the diameter of ophthalmic artery (the

fellow healthy eye), *control OA* *D* the diameter of ophthalmic artery in control group, *NAION OA* Vm the average

blood flow velocity of ophthalmic artery in the affected side of NAION patients, *normal OA Vm* the average blood

flow velocity of ophthalmic artery in the unaffected side of NAION patients, *control OA Vm* the average blood flow

velocity of ophthalmic artery in control group, *NAION OA vol* the blood flow volume of ophthalmic artery (NAION

eye), *normal OA vol* the blood flow volume of ophthalmic artery (the fellow healthy eye), *control OA vol*  the blood

flow volume of ophthalmic artery in control group

**supplementary data part 2**


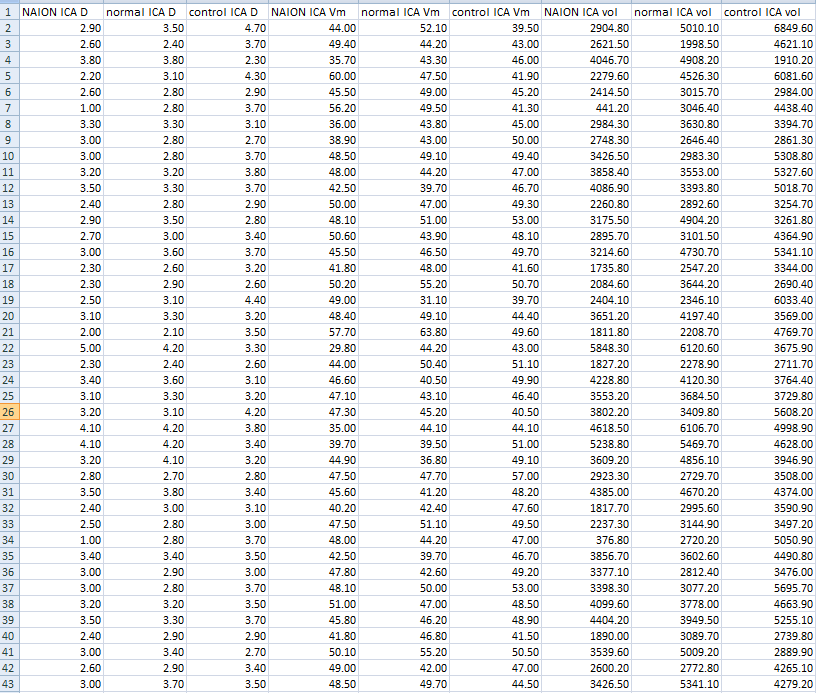


*NAION ICA D* the diameter of internal carotid artery in the affected side of NAION patients*, normal ICA D* the

diameter of internal carotid artery in the unaffected side of NAION patients, *control ICA D* the diameter of internal

carotid artery in control group, *NAION ICA Vm* the average blood flow velocity of internal carotid artery in the

affected side of NAION, *normal ICA Vm* the average blood flow velocity of internal carotid artery in the unaffected

side of NAION patients*, control ICA Vm* the average blood flow velocity of internal carotid artery in control group,

*NAION ICA vol* the blood flow volume of internal carotid artery in the affected side of NAION patients, *normal ICA*

*Vol* the blood flow volume of internal carotid artery in the unaffected side of NAION patients, *control ICA vol* the

blood flow volume of internal carotid artery in control group

**supplementary data part 3**


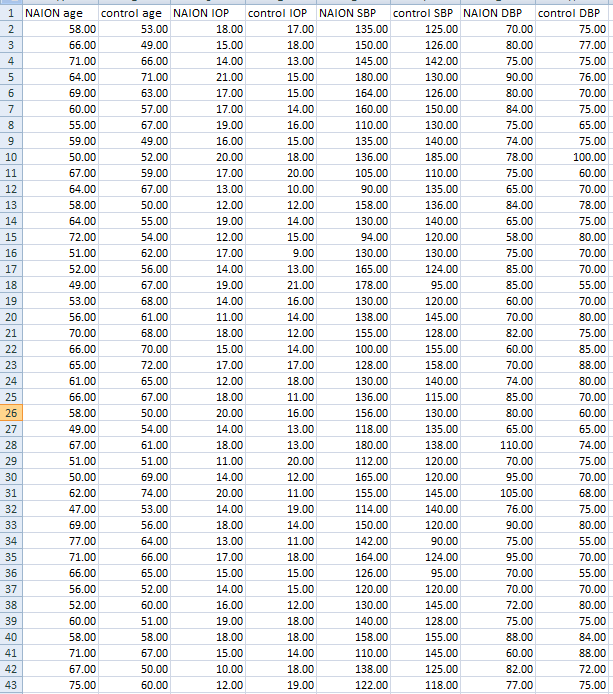


*NAION age* age of NAION patients, *control age* age of control group, *NAION IOP*  intraocular pressure of NAION

Patients, *control IOP* intraocular pressure of control group, *NAION SBP* systolic blood pressure of NAION

patients, *control SBP* systolic blood pressure of control group, *NAION DBP* diastolic blood pressure of NAION

patients, *control DBP* diastolic blood pressure of control group

**supplementary data part 4**


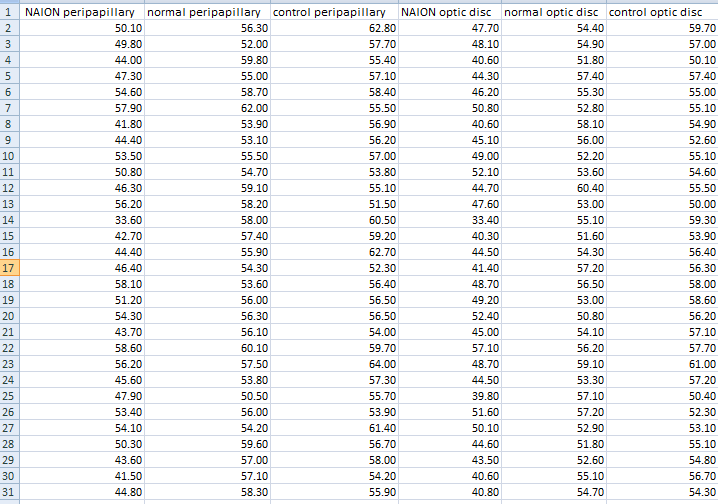


*NAION peripapillary* peripapillary vessel density in the affected side of NAION patients，*normal peripapillary*

peripapillary vessel density in the unaffected side of NAION patients，*control peripapillary* peripapillary vessel

density in control group，*NAION optic disc* optic disc vessel density in the affected side of NAION patients，

*normal optic disc*  optic disc vessel density in the unaffected side of NAION patients，*control optic disc* optic disc

vessel density in control group
